# Identifying and Exploring the Candidate Susceptibility Genes of Cirrhosis Using the Multi-Tissue Transcriptome-Wide Association Study

**DOI:** 10.3389/fgene.2022.878607

**Published:** 2022-05-13

**Authors:** Xiao-Bo Zhu, Yu-Qing Hou, Xiang-Yu Ye, Yi-Xin Zou, Xue-Shan Xia, Sheng Yang, Peng Huang, Rong-Bin Yu

**Affiliations:** ^1^ The People’s Hospital of Danyang, Affiliated Danyang Hospital of Nantong University, Zhenjiang, China; ^2^ Department of Epidemiology, Center for Global Health, School of Public Health, Nanjing Medical University, Nanjing, China; ^3^ Faculty of Life Science and Technology, Kunming University of Science and Technology, Kunming, China; ^4^ Department of Biostatistics, Center for Global Health, School of Public Health, Nanjing Medical University, Nanjing, China

**Keywords:** transcriptome-wide association study, cirrhosis, multiple tissues, genome-wide association studies, gene expression

## Abstract

**Objective:** We identify and explore the candidate susceptibility genes for cirrhosis and their underlying biological mechanism.

**Methods:** We downloaded the genome-wide association studies summary data of 901 cirrhosis cases and 451,363 controls and integrated them with reference models of five potential tissues from the Genotype-Tissue Expression (GTEx) Project, including whole blood, liver, pancreas, spleen, and thyroid, to identify genes whose expression is predicted to be associated with cirrhosis. Then, we downloaded gene expression data of individuals with hepatocellular carcinoma from TCGA database to conduct differential expression analysis to validate these identified genes and explored their possible role in driving cirrhosis via functional enrichment and gene set enrichment analysis (GSEA).

**Results:** We identified 10 significant genes (*SKIV2L*, *JPH4*, *UQCC2*, *RP11-91I8.3*, *MAU2*, *ERAP1*, *PUS3*, *ZNF677*, *ARHGAP40*, and *SHANK3*) associated with cirrhosis at a Bonferroni-corrected threshold of *p* < 0.01, among which two (*SKIV2L* and *JPH4*) were identified in the liver and five (*SKIV2L*, *JPH4*, *MAU2*, *SHANK3*, and *UQCC2*) were validated by differential expression analysis at an FDR-corrected threshold of *p* < 0.01. The enrichment analysis showed that the degradation process of RNA, which is enriched by 58 genes, is significantly under-enriched in liver cancer tissues (*p* = 0.0268).

**Conclusion:** We have identified several candidate genes for cirrhosis in multiple tissues and performed differential genetic analysis using the liver cancer database to verify the significant genes. We found that the genes *SKIV2L* and *JPH4* identified in the liver are of particular concern. Finally, through enrichment analysis, we speculate that the process of mRNA transcription and RNA degradation may play a role in cirrhosis.

## Introduction

Cirrhosis is a diffuse liver disease characterized by fibrosis and structural abnormalities due to different mechanisms of liver injury (Tsochatzis et al., 2014; Fukui et al., 2016; Garcia-Tsao et al., 2017). The progression and outcome of cirrhosis are very serious. Cirrhosis will increase the risk of hepatocellular carcinoma (HCC) and will ultimately lead to death inevitably (Tsochatzis et al., 2014; Villanueva, 2019). Studies have shown that cirrhosis is the 14th most common cause of death in adults worldwide (Lozano et al., 2012). In addition, it can also cause serious complications that worsen the prognosis of cirrhosis (Fukui et al., 2016). Cirrhosis may also involve multiple organs throughout the body (Arroyo et al., 2021). Cirrhosis is known to cause changes in the blood (Bajaj et al., 2019); patients with cirrhosis have symptoms of splenomegaly in the advanced stage (Fofiu et al., 2021), and studies have shown that patients with liver dysfunction also suffer from pancreatic dysfunction (Epstein et al., 1982). Furthermore, according to our previous studies, there is a correlation between cirrhosis and thyroid dysfunction (Huang et al., 2021).

Many studies have explored the risk factors of cirrhosis, mainly including heavy alcohol intake (Roerecke et al., 2019), chronic viral hepatitis B and C infections (Mokdad et al., 2014), autoimmune hepatitis (Basyte-Bacevice et al., 2019), and a growing obesity epidemic (Mokdad et al., 2014). Research studies have also shown that the occurrence of cirrhosis is related to the genetic background of the host (Fabris et al., 2009; Kupcinskas et al., 2017; Basyte-Bacevice et al., 2019). In recent years, genome-wide association studies (GWAS) have identified a number of susceptibility loci (Innes et al., 2020; Emdin et al., 2021). However, most of the identified variants are located in non-coding regions, which make their functional characterization of disease occurrence inaccurate and complicated (Maurano et al., 2012). In addition, complicated linkage disequilibrium (LD) may obscure the causal variation leading to the association. These challenges have prompted the development of GWAS to map susceptibility genes and new analytical methods (Tam et al., 2019).

The transcriptome-wide association study (TWAS) allows us to explore the genetic basis underlying complicated diseases such as cirrhosis from the perspective of gene expression, by integrating GWAS data and external expression quantitative trait loci (eQTL) data (Bossé et al., 2020). TWAS uses one or more constructed eQTL reference panels to predict gene–trait associations (Wainberg et al., 2019). To date, many TWAS analyses have been applied to the identification of candidate genes for complicated diseases, such as mental disorders (Derks and Gamazon, 2020), pancreatic cancer (Liu et al., 2020), lung cancer (Zhu et al., 2021), and pulmonary fibrosis (Gong et al., 2020) and provided many valuable clues for a better understanding of the occurrence and progression of diseases. However, for many complicated traits, the biologically relevant tissues are unknown. Most existing studies identify gene–trait associations based on a single tissue, and the significant gene effects identified thereby are exaggerated. Also, studies have shown that eQTL with larger effect tends to regulate gene expression in multiple tissues. TWAS analysis in multiple tissues can improve the accuracy of the results.

In this study, we employed TWAS to explore the cirrhosis-related genes. Considering cirrhosis is associated with multiple tissues, we used reference panels of eQTL for five tissues, including whole blood, liver, pancreas, spleen, and thyroid, together with GWAS summary data on cirrhosis to identify tissue-specific susceptibility genes. Meanwhile, we also used differential gene expression analysis and functional enrichment analyses to validate the identified susceptibility genes. Our study might pave the way to further reveal the mechanism underlying cirrhosis.

## Methods

### GWAS Summary Data

We obtained the GWAS summary data of cirrhosis (*n* = 452,264, prevalence = 1.99%) from the Gene ATLAS website (http://geneatlas.roslin.ed.ac.uk/), a large database of associations between hundreds of traits and millions of variants using White British individuals for the UK Biobank (Canela-Xandri et al., 2018). The association statistics was calculated under a linear mix model, with sex, array batch, UK Biobank Assessment Center, age, age2 (Canela-Xandri et al., 2018), and the top 20 genotype PCs included as fixed effects. Following Marees et al., (2018), we filtered out variants with MAF <0.01 or HWE *p*-value <1e−10. Finally, we used *LDSC* (version 1.0.1) to transform the filtered summary data into the LD-score format as well as computed the heritability.

### Transcriptome-Wide Association Analysis of Multiple Tissues

*FUSION* was applied to the formatted GWAS summary data for tissue-related TWAS analysis, including whole blood, liver, pancreas, spleen, and thyroid (Gusev et al., 2016). Briefly, *FUSION* uses precomputed gene expression weights, along with disease GWAS summary statistics, to calculate the association of each gene with disease. The association statistics was defined as TWAS Z-score and estimated as following:

$${\text{Z}}_{\text{TWAS}}\;=\text{WZ}/{\left({\text{W Σ}}_{\text{s},\text{s}}{\text{ W}}^{\text{t}}\right)}^{1/2}.$$

${\text{Z}}_{\text{TWAS}}$ has a distribution that depends both on Z and the weights W_._ ${\text{Z}}_{\text{TWAS}}$ is well calibrated (has a mean of 0 and unit variance) only under the null model of Z ∼ N (0,Σs,s), where Z is the estimation of SNP on the target disease, W is the weights, and S is the covariance among all SNPs (i.e., linkage disequilibrium). We used the LD reference panel of Europeans from the 1,000 Genome Project (Speed et al., 2020). For each tissue, we downloaded the GTEx V7-based functional weights precomputed by *FUSION* (http://gusevlab.org/projects/fusion/). *FUSION* computed the SNP-expression weights within the 1 Mb window of a given gene by the best expression prediction models among top eQTL, the best linear predictor (BLUP) (Gusev et al., 2016), the Bayesian linear mixed model (BSLMM) (Zhou et al., 2013), least absolute shrinkage and selection operator (LASSO) (Tibshirani, 1996; Veturi and Ritchie, 2018), and elastic net (Zou and Hastie, 2005), depending on the highest cross-validation R^2^. Specifically, we obtained five reference eQTL data. The sample size of the five-reference eQTL data is 153 (liver), 220 (pancreas), 146 (spleen), 399 (thyroid), and 369 (whole blood). The number of genes of the five-reference eQTL data is 2,914 (liver), 5,094 (pancreas), 4,497 (spleen), 9,826 (thyroid), and 6,007 (whole blood). We also applied the Bonferroni correction to account for multiple hypotheses and set the *p*-value threshold at 0.01.

### TCGA Data Processing and Differential Expression Analysis

We downloaded gene expression data (HTSeq-Counts) and corresponding clinical information of 377 individuals with hepatocellular carcinoma from TCGA database (Adrian Ally et al., 2017), retained those with paired adjacent tissue samples, and obtained data with 100 samples (50 tumor and 50 adjacent tissue samples). In quality control steps, we first mapped the ENSEMBL ID to gene symbol ID and averaged those expressions that mapped to the same symbol. Then, we filtered out genes expressed in fewer than three samples. Finally, we retained the expression matrix with 100 samples and 13,124 genes and applied TMM normalization to the filtered matrix. We used the *limma* package (version 3.50.0) to conduct differential expression analysis (Ritchie et al., 2015). Gene with | log_2_ (fold change) |≥1 and false discovery rates (FDR)-adjusted *p*-value<0.01 was defined as differentially expressed genes (DEGs).

### Pathway and Gene Set Enrichment Analyses

We used the *clusterProfiler* package (version 4.0.5) to perform Gene Ontology (GO) and Kyoto Encyclopedia of Genes and Genomes (KEGG) enrichment analyses on cirrhosis-related genes identified by TWAS. Considering the small number of genes to be analyzed, we set a relatively relaxed significance threshold of FDR<0.1. In addition, we used the *clusterProfiler* package to perform gene set enrichment analysis (GSEA) on differentially expressed genes in HCC samples versus adjacent samples. The pathway reached a threshold of FDR<0.05 was considered significant.

## Results

### 3.1 Multi-Tissue Transcriptome-Wide Significant Genes

**FIGURE 1:**
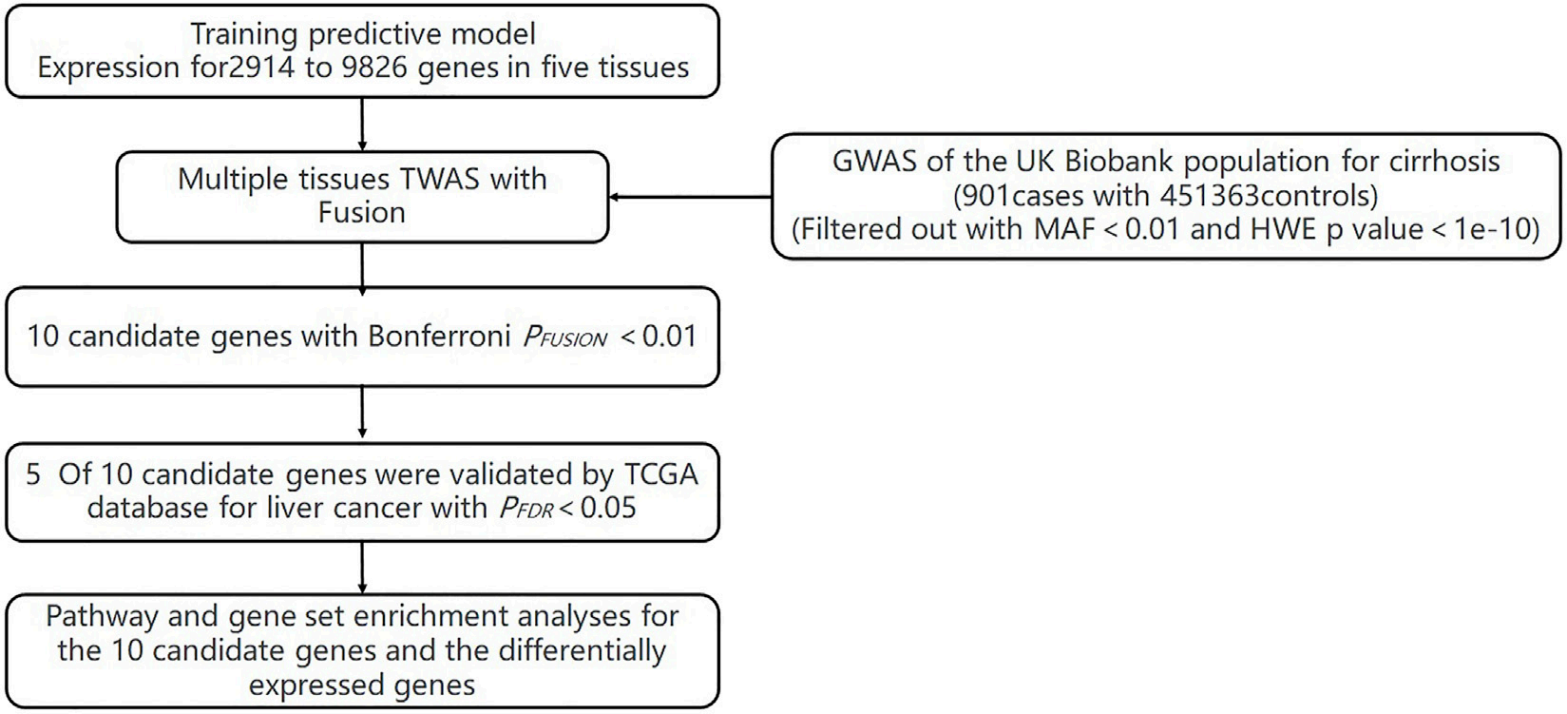
Flowchart of the study.

**FIGURE 2:**
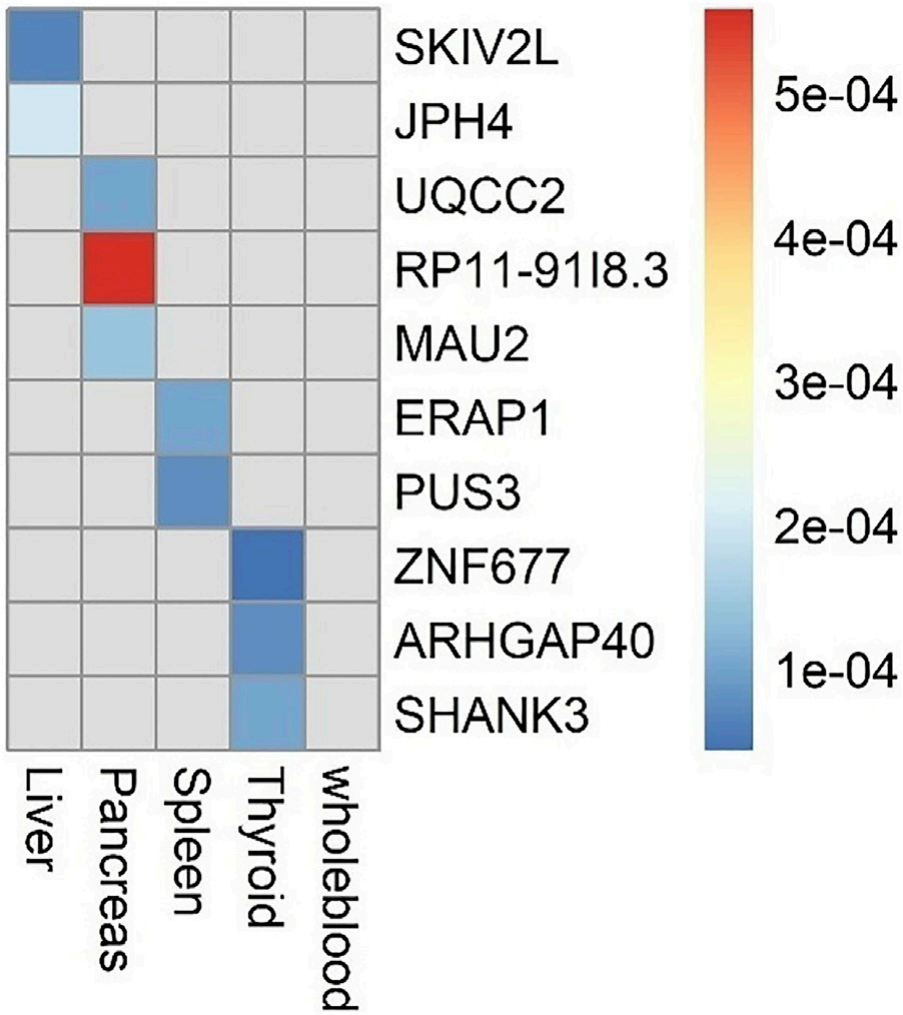
Genes significantly associated with risk of cirrhosis in each tissue.

The flowchart of the study is shown in Supplementary Material (Figure 1). We evaluated the association between predicted gene expression and cirrhosis using *FUSION*. Totally, we found that ten genes across four tissues were significantly associated with cirrhosis after the Bonferroni correction (Figure 2; Table 1, Supplementary Table S1). Specifically, in liver tissue, we defined *SKIV2L* at 6p21.33 (*P*_*adjusted*_ = 0.011) and *JPH4* at 14q11.2 (*P*_*adjusted*_ = 0.019) as cirrhosis-related genes, while in pancreas tissue, we found *UQCC2* at 6p21.31 (*P*_*adjusted*_ = 0.027), RP11-91I8.3 (*P*_*adjusted*_ = 0.041), and *MAU2* at 19p13.11 (*P*_*adjusted*_ = 0.047) significantly associated with cirrhosis. Other significant genes included *ERAP1* at 5q15 (*P*_*adjusted*_ = 0.019) and *PUS3* at 11q24.2 (*P*_*adjusted*_ = 0.018) in spleen tissue and *ZNF677* at 19q13.42 (*P*_*adjusted*_ = 0.029), *ARHGAP40* at 20q11.23 (*P*_*adjusted*_ = 0.017), and *SHANK3* at 22q13.33 (*P*_*adjusted*_ = 0.030) in thyroid tissue. We did not define genes expressed in whole blood as significantly associated with cirrhosis.

**TABLE 1:** Genes significantly associated with risk of cirrhosis.

Tissue	Gene	CHR	MODEL	TWAS.Z	TWAS.P	*P-Bonferroni*
Liver	SKIV2L	6	blup	−3.9944	6.49E-05	0.011293
Liver	JPH4	14	blup	−3.7205	0.000199	0.018507
Pancreas	UQCC2	6	blup	3.88417	0.000103	0.02678
Pancreas	RP11-91I8.3	18	enet	−3.4513	0.000558	0.040734
Pancreas	MAU2	19	top1	3.797	0.000146	0.047012
Spleen	ERAP1	5	lasso	3.884217	0.000103	0.019364
Spleen	PUS3	11	top1	3.96	7.49E-05	0.018425
Thyroid	ZNF677	19	top1	4.088	4.35E-05	0.029145
Thyroid	ARHGAP40	20	lasso	−3.96114	7.46E-05	0.017233
Thyroid	SHANK3	22	top1	−3.878	0.000105	0.02982

CHR: the chromosome on which the identified gene is located.

MODEL: models used for imputation in *FUSION*.

TWAS.P: *p*-values for TWAS analysis in each tissue.

P-Bonferroni: *p*-values corrected by Bonferroni for TWAS analysis in each tissue.

### TWAS Validation Using the Gene Expression Profile of Liver Cancer

We used RNA-seq data from TCGA to explore genes whose expression was significantly associated with HCC. With the threshold of | log_2_ (fold change) |≥1 and FDR-adjusted *p*-value<0.01, we totally defined 7,163 differentially expressed genes in HCC samples versus adjacent samples, including 1,631 upregulated genes and 5,532 downregulated genes (Supplementary Table S2). Notably, five genes identified by TWAS overlapped with these DEGs, four of which were significantly downregulated, including *UQCC2*, *MAU2*, *SHANK3*, and *SKIV2L*, while the remaining one, *JPH4*, was significantly upregulated (Supplementary Table S3).

### Functional Enrichment Analyses

**FIGURE 3:**
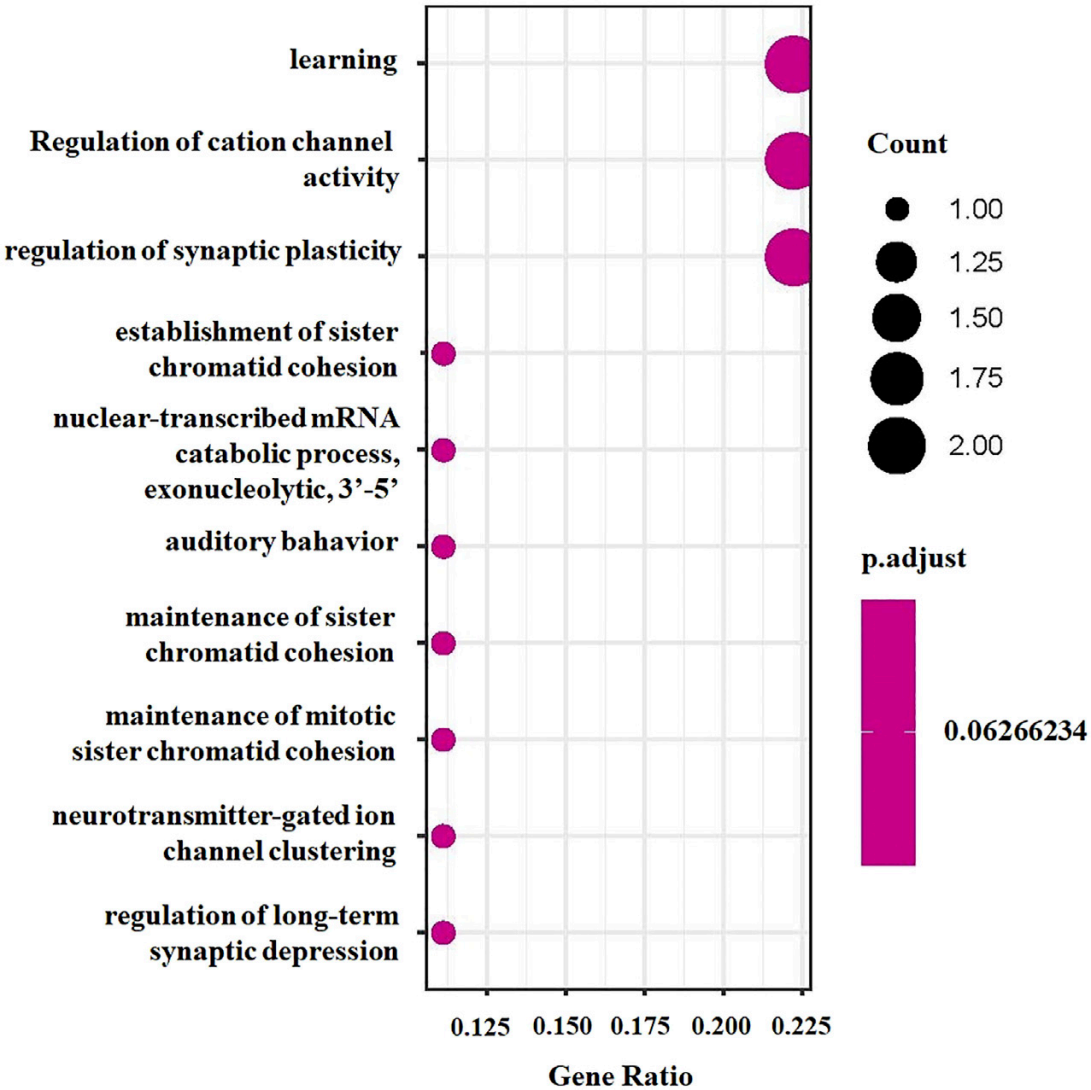
GO enrichment analysis bubble chart for significant genes.

To explore the potential biological mechanisms underlying the identified associations, we first applied functional enrichment analyses to cirrhosis-related genes identified by TWAS. We found that these 10 genes were significantly enriched in several GO terms, including learning and nuclear-transcribed mRNA catabolic process, exonucleolytic, 3′-5' (Figure 3), and three KEGG pathways, including RNA degradation, glutamatergic synapse, and herpes simplex virus type 1 infection (Supplementary Figure S1). Then, we performed GSEA based on the differentially expressed gene sets between cancer and adjacent samples and obtained 631 GO terms (Supplementary Table S4) and significantly differentially enriched 88 KEGG pathways (Supplementary Table S5). We found that learning and nuclear-transcribed mRNA catabolic process, exonucleolytic, and 3′-5′ were among the differentially enriched GO terms (Supplementary Figure S2), while RNA degradation and herpes simplex virus type 1 infection were among the differentially enriched KEGG pathways (Supplementary Figure S3).

## Discussion

In this study, we used tissue-specific TWAS to explore the association of genetically predicted gene expression with cirrhosis in European ancestry and identified 10 genes in four tissues as potential cirrhosis-related genes, including two in the liver (*SKIV2L* and *JPH4*), three in the pancreas (*UQCC2*, *RP11-91I8.3*, and *MAU2*), two in the spleen (*ERAP1* and *PUS3*), and three in thyroid tissue (*ZNF677*, *ARHGAP40*, and *SHANK3*). The aforementioned ten genes have never been clearly reported to be associated with cirrhosis. In addition, the expression of five of the 10 identified genes, including *JPH4*, *UQCC2*, *MAU2*, *SHANK3*, and *SKIV2L*, also showed significant changes in HCC samples compared to the adjacent liver samples.

*SKIV2L* is located at 6p21.33 and encodes a gene for helicase, which is a component of the SKI complex (Yang et al., 1998). The SKI complex is thought to be involved in exosome-mediated RNA decay and associated with transcriptionally active genes (Synowsky and Heck, 2008). Genetic variations in *SKIV2L* are known to be associated with the occurrence of trichohepatoenteric syndrome (Vardi et al., 2018), an autosomal recessive genetic disease that may cause immune dysfunction and liver abnormalities (Fabre et al., 1993). A number of GWAS have shown that *SKIV2L* is associated with the risk of chronic hepatitis B virus infection (Jiang et al., 2015) and chronic hepatitis C virus infection (Sakaue et al., 2021), two major causes of cirrhosis (Bedossa, 2015) as well as liver damage (Nicoletti et al., 2019) and liver tumors (Sakaue et al., 2021). Experimental studies have shown that *SKIV2L*, a member of the NSDK, could specifically regulate the production of type I interferon (Zhou et al., 2019). It may fine-tune antiviral responses through RIG1/MDA5 to prevent excessive inflammation that may lead to tissue damage (Eckard et al., 2014). Therefore, deletion or partial functional deficiency of *SkIV2L* may cause uncontrolled or dysregulated production of RLR ligands or sensors resulting in increased autoinflammatory responses in the absence of viral infection or increased susceptibility to systemic autoimmune diseases (Fernando et al., 2007; McKay et al., 2009; Yoneyama et al., 2014). Another cirrhosis-related gene, *JPH4*, found in liver tissue, is located at 14q11.2. *JPH4* is previously reported as brain-specific and appears to have an active role in certain neurons involved in motor coordination and memory (Hogea et al., 2021). Another research showed that *JPH4* is associated with the prognosis of renal cell carcinoma (Zhang et al., 2020). At present, there are few research reports on the *JPH4* gene and diseases. Efforts may have to be made to validate its role in liver-related diseases.

In addition, we need to pay attention to the significant genes *MAU2* and *UQCC2* in pancreatic tissue and the significant gene *SHANK3* in thyroid tissue. The results of our analysis show that these three genes are all under-expressed in liver cancer tissue. They are located at 19p13.11, 6p21.31, and 22q13.33, respectively. MAU2 is a regulatory subunit that constitutes cohesin, a highly conserved protein complex that plays an important role in sister chromatid cohesion, chromatin structure, gene expression, and DNA repair (Minina et al., 2017). Recent studies have shown that the gene encoding the cohesin subunit is somatically mutated in a variety of human cancers (Hill et al., 2016). Knockdown of *CDCA5*, another regulatory subunit of cohesin, can induce liver cancer (Chen et al., 2019). The results of our study may also reveal the role of the cohesin subunit of *MAU2* in liver cancer. *UQCC2*, another prominent gene in pancreatic tissue, is a nuclear-encoded gene for respiratory chain complex III subunits. Studies have shown that mutations in coding genes can lead to respiratory complex III deficiency (Lonlay et al., 2001), which in turn leads to liver failure (Iwama et al., 2011). Therefore, it can be speculated that abnormal expression of the *UQCC2* gene can lead to liver damage and cirrhosis by affecting the metabolic process of the respiratory chain. Given that *UQCC2* is a downregulated gene in liver cancer tissue, it can be speculated that the damage caused by this metabolism contributes to the occurrence of liver cancer. There is also a significant gene in thyroid tissue, i.e., *SHANK3*, which is also under-expressed in liver cancer tissue. However, research on *SHANK3* has mainly focused on the occurrence of autism (Uchino and Waga, 2015; Li et al., 2018), with little mention of the liver. As far as we know, in the advanced stage of cirrhosis, hepatic encephalopathy may occur, which is manifested as cerebral confusion. Therefore, we do not know the connection between the process of cirrhosis and the brain, which is an interesting association and worth exploring.

In addition to the aforementioned genes validated in liver cancer tissues, we also identified four candidate genes (*ZNF677*, *ARHGAP40*, *ERAP1*, and *PUS3*) in thyroid and spleen tissues, of which *ERAP1* has been reported to be associated with the development of sclerosing cholangitis, a disease that may progress to cirrhosis (Ellinghaus et al., 2016). Other genes have also been demonstrated to be associated with cancers. For example, *ZNF677* has been reported to be associated with the occurrence of non-small-cell lung cancer (Heller et al., 2015) and thyroid cancer (Siraj et al., 2021); *ARHGAP40* may affect the development of laryngeal squamous cell carcinoma (Wang et al., 2021); the splicing event of the *PUS3* gene at 11q24.2 has also been reported to be involved in a variety of cancers (Ajiro et al., 2016). Though few studies on these three genes and liver-related diseases are available now, the potential association may deserve more attention in the future research study.

Functional enrichment analysis found that the 10 genes identified by TWAS were significantly enriched in the catabolism process of nuclear-transcribed mRNA, and the GSEA results of the expression data of liver cancer tissue and adjacent tissue in this study also suggested that the set of genes for the degradation process of transcribed mRNAs is under-expressed in liver cancer tissues. This suggests that the occurrence of cirrhosis may be related to the transcriptional process of susceptibility genes.

There are some limitations to this study. First, our results were only been validated for differential genes in the liver cancer dataset and lacked revalidation results on other liver cirrhosis datasets. Moreover, there is still a certain gap between the disease pathology of liver cirrhosis and liver cancer, which may make our results less specific for liver cirrhosis. Also, the mechanism of susceptibility genes leading to the development of liver pathology is still unclear and needs further exploration. Second, this study is a multi-tissue TWAS analysis; however, there is no overlap of susceptibility genes identified in different tissues. In particular, susceptibility genes in non-liver tissue were not identified in liver tissue as well. Therefore, the results of TWAS must be interpreted with caution for cross-tissue results, especially for genes that are significant in other tissues.

In conclusion, our results present a list of genes that have not been previously focused on cirrhosis, some of which were also validated by differential expression analysis. Functional enrichment analysis, combined with previous knowledge of these identified genes, implies that they may contribute to cirrhosis through the transcriptional process of susceptibility genes and the process of RNA degradation. Our study may further expand our understanding of the pathogenesis of cirrhosis and hepatocellular carcinoma.

## Data Availability

Publicly available datasets were analyzed in this study. These data can be found at: GWAS summary data for cirrhosis (http://geneatlas.roslin.ed.ac.uk/), LD reference panel of European from the 1,000 Genome Project (https://data.broadinstitute.org/alkesgroup/FUSION/LDREF.tar.bz2), the GTEx V7-based functional weights for each tissue (https://gtexportal.org/home/), and gene expression data for hepatocellular carcinoma (https://www.cancer.gov/about-nci/organization/ccg/research/structural-genomics/tcga).
