# Supplementary material for: Identifying and Exploring the Candidate Susceptibility Genes of Cirrhosis Using the Multi-Tissue Transcriptome-Wide Association Study
Source: Front Genet. 2022 May 13;13:878607. doi: 10.3389/fgene.2022.878607 (PMC9136150; doi:10.3389/fgene.2022.878607)

**Supplementary Figure 1 KEGG pathway analysis bubble chart for significant genes**


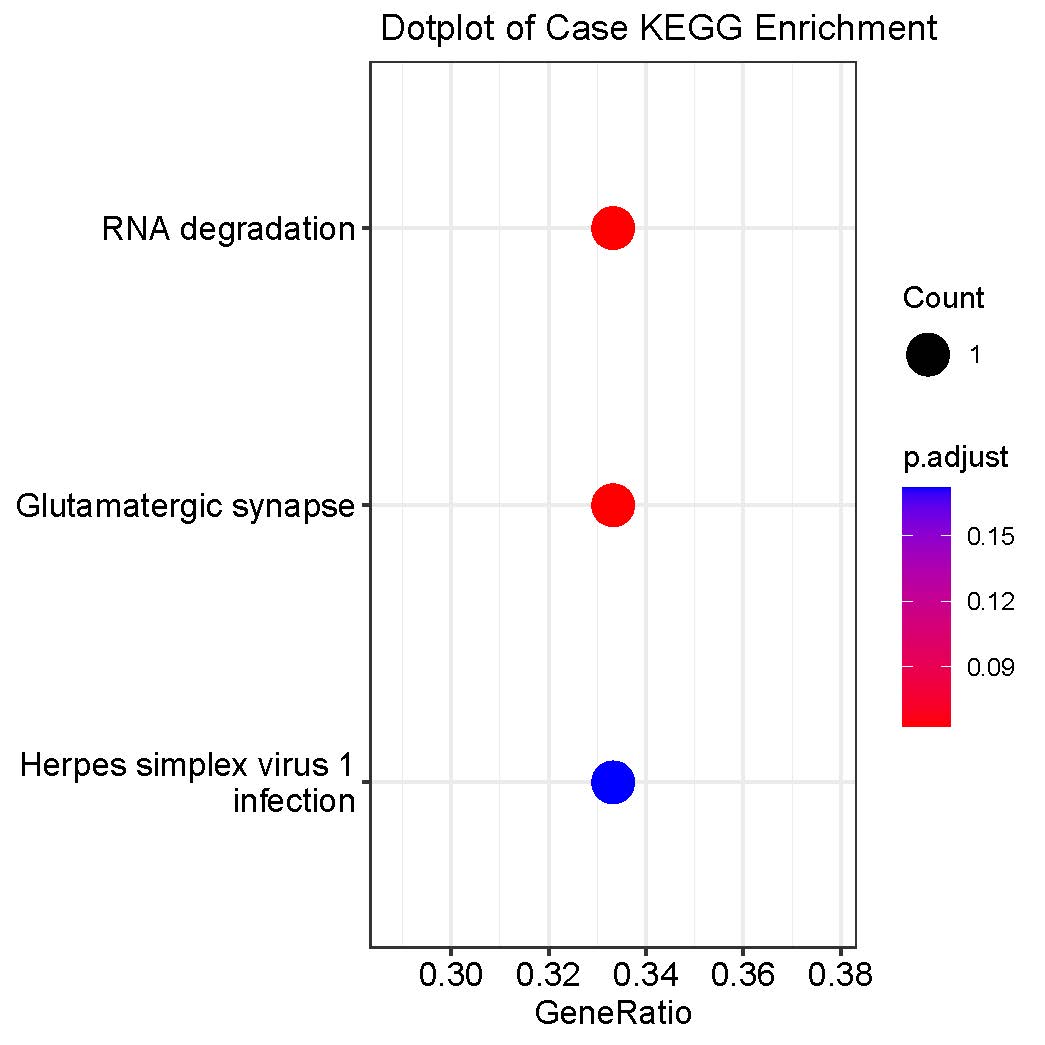


**Supplementary Figure 2: Enrichment plot: nuclear-transcribed mRNA catabolic process, exonucleolytic, 3'-5' _Go terms & learning _Go terms**

**Profile of the Runnig ES Score & Positions of Members on the Rank Ordered List**


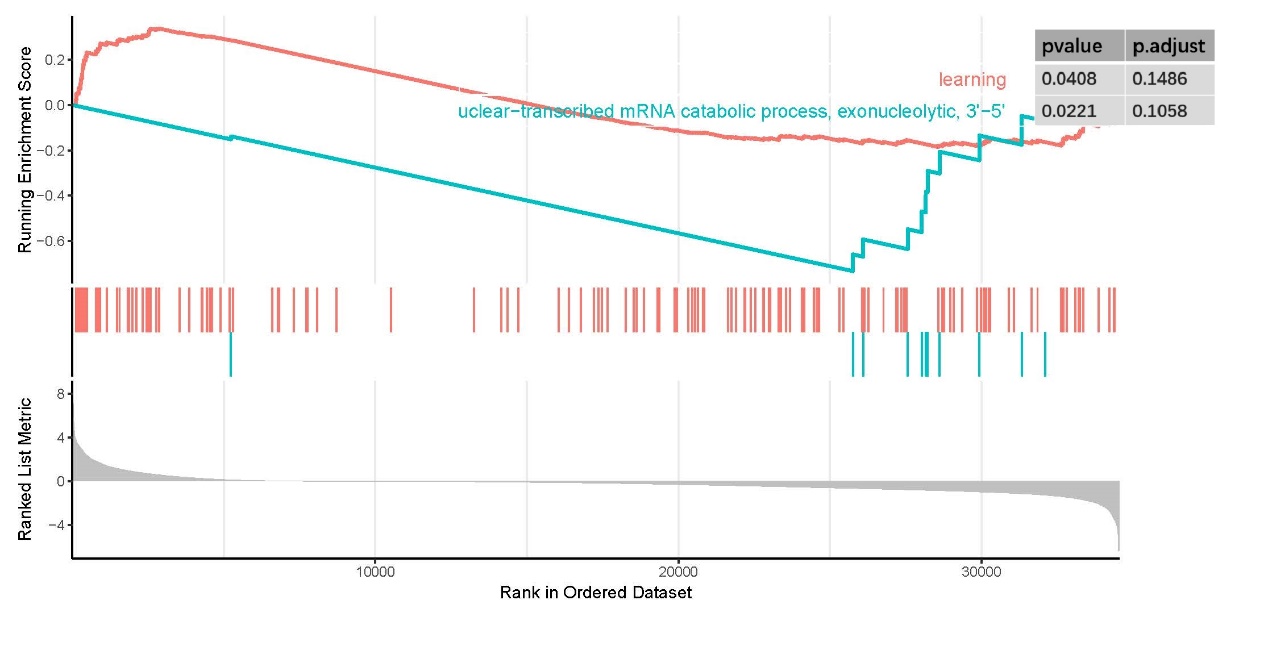


**Supplementary Figure 3: Enrichment plot: Herpes simplex virus 1 infection_PATHWAY & RNA degradation_ PATHWAY**

**Profile of the Runnig ES Score & Positions of Members on the Rank Ordered List**


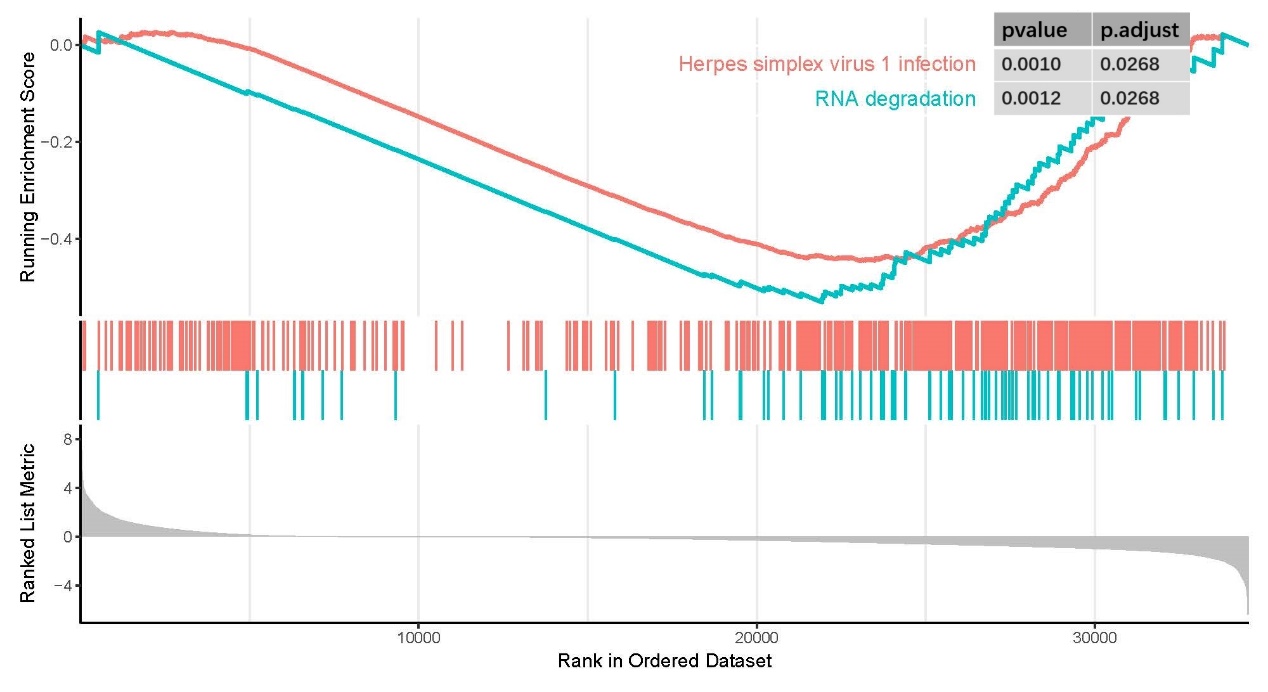

Supplement: Supplementary file 6 [file DataSheet1.docx]
